# Palliation of Gastric Outlet Obstruction in Case of Biliary Obstruction—A Retrospective, Multicenter Study: The B-GOOD Study

**DOI:** 10.3390/cancers16193375

**Published:** 2024-10-02

**Authors:** Alessandro Fugazza, Marta Andreozzi, Cecilia Binda, Andrea Lisotti, Ilaria Tarantino, Juan J. Vila, Carlos Robles Medranda, Arnaldo Amato, Alberto Larghi, Enrique Perez Cuadrado Robles, Giovanni Aragona, Francesco Di Matteo, Roberta Badas, Cesare Hassan, Carmelo Barbera, Benedetto Mangiavillano, Stefano Crinò, Matteo Colombo, Carlo Fabbri, Pietro Fusaroli, Antonio Facciorusso, Andrea Anderloni, Marco Spadaccini, Alessandro Repici

**Affiliations:** 1 Division of Gastroenterology and Digestive Endoscopy, Humanitas Research Hospital-IRCCS, 20089 Rozzano, Italy; alessandro.fugazza@humanitas.it (A.F.); marta.andreozzi@humanitas.it (M.A.); cesare.hassan@hunimed.eu (C.H.); matteo.colombo@humanitas.it (M.C.); marco.spadaccini@humanitas.it (M.S.); alessandro.repici@hunimed.eu (A.R.); 2 Gastroenterology and Digestive Endoscopy Unit, Forlì-Cesena Hospitals, AUSL Romagna, 47121 Forlì, Italy; cecilia.binda@gmail.com (C.B.); carlo.fabbri@auslromagna.it (C.F.); 3 Gastroenterology Unit, Hospital of Imola, University of Bologna, 40026 Imola, Italy; lisotti.andrea@gmail.com (A.L.); pietro.fusaroli@unibo.it (P.F.); 4 Endoscopy Service, Department of Diagnostic and Therapeutic Services, IRCCS-ISMETT, 90127 Palermo, Italy; itarantino74@gmail.com; 5 Endoscopy Unit, Gastroenterology Department, Hospital Universitario de Navarra, 310000 Navarra, Spain; juanjvila@gmail.com; 6 Ecuadorian Institute of Digestive Diseases (IECED), Guayaquil 090505, Ecuador; carlosoakm@yahoo.es; 7 Digestive Endoscopy and Gastroenterology Department, ASST Lecco, 23900 Lecco, Italy; arnamato@gmail.com; 8 Digestive Endoscopy Unit, Fondazione Policlinico Universitario A. Gemelli IRCCS, Università Cattolica del Sacro Cuore, 00136 Rome, Italy; alberto.larghi@yahoo.it; 9 Georges Pompidou European Hospital-APHP, 75015 Paris, France; enrique.perez.cr@gmail.com; 10 Gastroenterology and Digestive Endoscopy Unit, Hospital of Piacenza, 29100 Piacenza, Italy; g.aragona@ausl.pc.it; 11 Digestive Endoscopy Unit, Campus Bio-Medico, University of Rome, 00128 Rome, Italy; dimatteo.francesco@gmail.com; 12 Digestive Endoscopy Unit, University Hospital, 09124 Cagliari, Italy; rbadas@aoucagliari.it; 13 Gastroenterology and Digestive Endoscopy Unit, Nuovo Ospedale Civile S. Agostino-Estense, 41126 Baggiovara di Modena, Italy; carmelo.barbera@gmail.com; 14 Gastrointestinal Endoscopy Unit, Humanitas Mater Domini, 21053 Castellanza, Italy; benedetto.mangiavillano@mc.humanitas.it; 15 Digestive Endoscopy Unit, The Pancreas Institute, University Hospital of Verona, 37126 Verona, Italy; stefanofrancesco.crino@aovr.veneto.it; 16 Gastroenterology Unit, Department of Medical Sciences, University of Foggia, 71122 Foggia, Italy; 17 Gastroenterology and Digestive Endoscopy Unit, IRCCS Foundation Policlinico San Matteo, 27100 Pavia, Italy; a.anderloni@smatteo.pv.it

**Keywords:** EUS-guided choledocoduodenostomy (EUS-CDS), distal malignant biliary obstruction (DMBO), gastric outlet obstruction (GOO), pancreatic cancer, enteral stenting (ES), EUS-guided gastroenterostomy (EUS-GE)

## Abstract

**Simple Summary:**

The aim of our retrospective study was to compare EUS-guided gastroenteroanastomosis (EUS-GE) and enteral stenting (ES) for the palliation of gastric outlet obstruction (GOO) in patients already treated with EUS-guided choledocoduodenostomy (EUS-CDS) for distal malignant biliary obstruction (DMBO). Our results on 77 patients demonstrated that both EUS-GE and ES are safe and effective for palliation of GOO, but EUS-GE is associated with less recurrence of symptoms.

**Abstract:**

Background: EUS-guided gastroenterostomy (EUS-GE) is a novel and effective procedure for the management of malignant gastric outlet obstruction (GOO) with more durable results when compared to enteral stenting (ES). However, data comparing EUS-GE to ES in patients already treated with EUS-guided choledocoduodenostomy (EUS-CDS) for distal malignant biliary obstruction (DMBO) are lacking. We aimed to compare outcomes of EUS-GE and ES for the palliation of GOO in this specific population of patients. Methods: A multicenter, retrospective analysis of patients with DMBO treated by EUS-CDS and subsequent GOO treated by EUS-GE or ES from 2016 to 2021 was conducted. Primary outcomes were overall AEs rate and dysfunction of the EUS-CDS after GOO treatment. Secondary outcomes included clinical success, technical success, procedure duration, length of hospital stay and relapse of GOO symptoms. Results: A total of 77 consecutive patients were included in the study: 25 patients underwent EUS-GE and 52 underwent ES. AEs rate and patency outcomes of the EUS-CDS after GOO treatment were comparable between the two groups (12.5% vs. 17.3%; *p* = 0.74). No recurrence of GOO symptoms was registered in the EUS-GE group while 11.5% of ES patients had symptoms recurrence, even if not statistically significant (*p* = 0.16), after a mean follow-up period of 63.5 days. Conclusion: EUS-GE and ES are both effective and safe for the palliation of GOO in patients already treated by EUS-CDS for DMBO with no difference in the biliary stent dysfunction rate and overall AEs. EUS-GE is associated with less recurrence of GOO symptoms.

## 1. Introduction

Gastric outlet obstruction (GOO) is an invalidating condition which worsens quality of life and reduces the therapeutic chances of patients affected by pancreatic, gastric, or duodenal cancer [1]. Frequently, GOO and distal malignant biliary obstruction (DMBO) coexist within the same patients, with jaundice usually showing up before GOO symptoms [2]. In the last years, EUS-guided drainages have been largely spreading, thanks to the advent of dedicated devices (i.e., lumen apposing metal stents—LAMS) [3] which are expanding our armamentarium for the treatment of both luminal and biliary issues [4,5,6,7]. 

**Figure 1:**
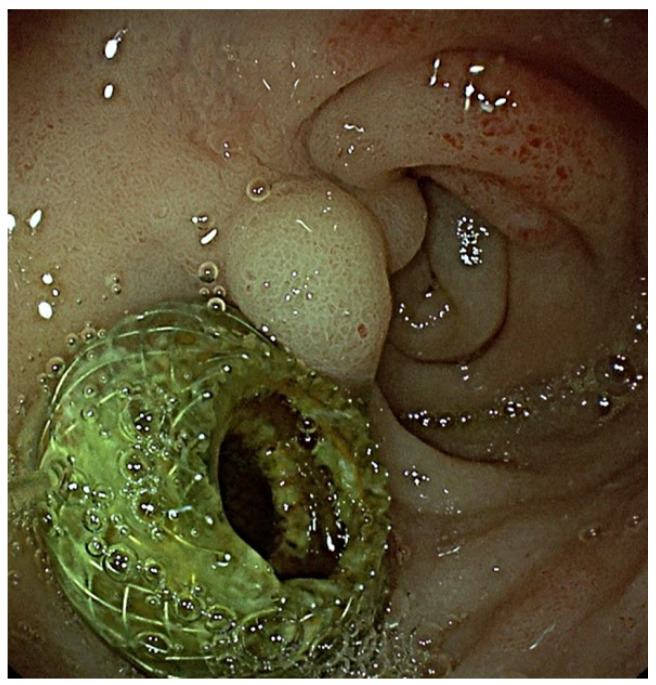
Endoscopic image of a duodenal stenosis in a patient already treated with EUS-BD for DMBO.

As a matter of fact, endoscopic ultrasound-guided choledocoduodenostomy (EUS-CDS) is to date considered a valuable option for biliary drainage in case of DMBO after ERCP failure, with high rate of both technical and clinical success and acceptable rate of adverse events (AEs) [8,9,10]. Figure 1.

On the other hand, endoscopic enteral stenting (ES) with self-expandable metal stents (SEMS) has been the main palliative strategy for GOO for many years [11,12]. Recently, EUS-guided gastroenterostomy (EUS-GE) has been introduced for the palliation of GOO, showing superior results in terms of stent patency and rate of reinterventions, compared to ES [13,14,15]. 

Nevertheless, the best therapeutic option is still not defined when GOO is associated to DMBO, especially when EUS-CDS has been performed. In this setting, EUS-CDS has been shown to be associated with a higher risk of dysfunction, and ascending cholangitis due to food impaction, and/or stent obstruction, when a duodenal stricture is present [16].

**Figure 2:**
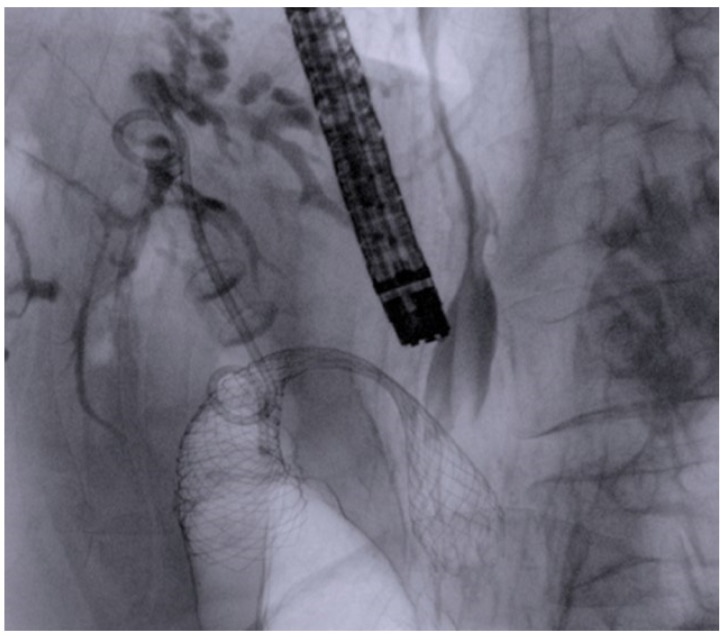
Fluoroscopic image of a LAMS positioned in the duodenal bulb for EUS-BD, with a double pig-tail plastic stent inside (stent-in-stent) and a duodenal metal stent positioned for concomitant duodenal stenosis in the same patient.

**Figure 3:**
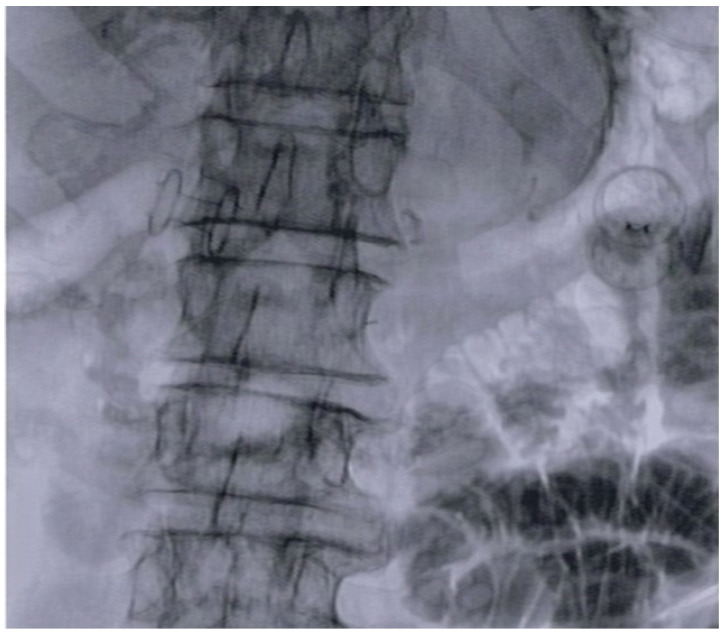
Fluoroscopic image of two LAMS positioned in the same patient for EUS-BD and EUS-GE for concomitant DMBO and duodenal stenosis.

The aim of our study was to collect data of all consecutive patients affected by DMBO drained using EUS-CDS, with concomitant or subsequent development of GOO treated by ES or EUS-GE, to evaluate and compare outcomes of the two different combinations. Figure 2 and Figure 3.

## 2. Materials and Methods

We conducted a retrospective, multicenter study. The study protocol was approved by the institutional review board (IRB) of each participating center and reported according to the STROBE checklist [17].

The site investigators and their research teams collected, reviewed, and entered the data into an electronic database (Redcap) maintained by the coordinating center. All authors had access to the study data and reviewed and approved the final manuscript.

From January 2016 to September 2021, all consecutive patients treated in 14 international centers by EUS-CDS for DMBO after ERCP failure, and subsequent GOO treated by EUS-GE or ES, were included. We excluded from the analysis underaged patients (<18 years old), patients under anti-thrombotic therapy or coagulopathy preventing invasive endoscopic therapy according ESGE guidelines [18], and patients unwilling or unable to sign the informed consent form.

### 2.1. Procedures

All procedures were performed with the patients under deep sedation with Propofol and Fentanyl or under general anesthesia in accordance with local sedation policies at the discretion of the anesthesiology. The procedures were performed either during the same or a different subsequent session of the EUS-CDS. In the case of same session, EUS-CDS was performed before EUS-GE or ES.

EUS-GE was performed using electrocautery-assisted LAMS (Hot-Axios, Boston Scientific). LAMS size was at the discretion of the endoscopists. The technique, balloon-assisted or freehand, also differed between the endoscopists according to their own experience [19,20]. The balloon-assisted method consists of passing a stone retrieval balloon or dilating balloon across the obstruction so that, after the inflation of the balloon with contrast fluid, the site of the puncture is facilitated by the visualization of the balloon under the EUS guide. The freehand technique entails the direct puncture of a small bowel loop adjacent to the gastric wall [21].

ES was performed using duodenal SEMS. SEMS type and size were at the discretion of the endoscopists [22].

### 2.2. Outcomes

Primary outcomes were (1) the rate of AEs classified according to the American Society for Gastrointestinal Endoscopy (ASGE) lexicon severity grading system [23]; (2) the rate of dysfunction of the EUS-CDS defined as the occurrence of biliary stent migration/obstruction, cholangitis, or jaundice occurring after GOO treatment; and (3) the need for re-intervention defined as the percentage of patients requiring additional endoscopic intervention due to biliary stent dysfunction. 

Secondary outcomes were (1) the rate of clinical success of GOO treatment, defined as the improvement in enteral diet assumption (soft solids, low residue, or full diet) comparing to the baseline according to the GOO scoring (≥1 point) [12]; (2) the rate of technical success defined as the ability to complete EUS-GE or ES with LAMS or SEMS placement, respectively; (3) duration of hospital stay; (4) procedural time; (5) the rate of dysfunction of the EUS-GE defined as the occurrence of stent migration, obstruction, or relapse of GOO symptoms.

### 2.3. Statistical Analysis

Descriptive statistical analysis was conducted on the available data. For normally distributed variables, means with standard deviations (SDs) were calculated, while variables with skewed distributions were presented as medians with interquartile ranges (IQRs). Categorical variables were expressed as frequencies and percentages.

## 3. Results

Seventy-seven consecutive patients (39, 74% males, mean age 70.17 years old) were enrolled over the study period. The most common etiology was pancreatic cancer (68–84.6%), followed by ampullary cancer (4–5.7%), and extra-hepatic cholangiocarcinoma (4–1.9%). All patients were treated by EUS-CDS for DMBO after ERCP failure, and the main reason was an unreachable papilla. 

The endoscopic procedures for GOO treatment were performed after a mean time of 14.3 ± 4.5 days from the EUS-CDS. Twenty-five (32.5%) patients were treated by EUS-GE and fifty-two (67.5%) patients were treated by ES. Patients’ demographics and baseline characteristics are summarized in Table 1.

### 3.1. Primary Outcomes

The AEs rate was comparable (*p* = 0.27) between EUS-GE (8%) and ES groups (17.3%). Specifically, one case of stent migration and one case of bleeding occurred in the EUS-GE group while three cases of bleeding, one case of stent obstruction, and five relapse of GOO symptoms were registered in the ES group. Moreover, no differences in term of EUS-CDS stent dysfunction (12.5% vs. 17.3%; *p* = 0.74) and rate of reintervention (12.5% vs. 23.1%; *p* = 0.36) were reported between the two groups.

### 3.2. Secondary Outcomes

Technical success in treating the GOO was achieved in 24 out of 25 (96%) patients in the EUS-GE group and all 52 patients (100%) in the ES group (*p* = 0.32). No significant differences were observed in the mean procedure duration time (30.0 ± 13.6 vs. 25.0 ± 12.1 min; *p* = 0.20) between the EUS-GE and ES groups. Procedural details of EUS-GE and ES are listed in Table S1 and Table S2, respectively.

Clinical success was achieved in all patients treated by EUS-GE and 98% of patients treated by ES (*p* = 1.0). The mean GOOS scores pre- and post-procedure were 0.88 vs. 2.6 in the EUS-GE group and 0.98 vs. 2.6 in the ES group, with a comparable GOOS score improvement (GE group + 1.72 points vs. ES group + 1,60 points; *p* = 0.46). Mean hospital stays (3 ± 2 vs. 5 ± 2 days; *p* = 0.15) were comparable between the two groups. Subclassifying AEs according to enteral stent patency, no significant difference was observed in terms of stent dysfunction rate (stent migration, stent obstruction, and relapse of GOO symptoms) between the two groups although a favorable trend was observed for EUS-GE (0% vs. 11.5 %, *p* = 0.16). Study outcomes are summarized in Table 2.

## 4. Discussion

Our multicenter experience shows that there is no incremental risk of biliary-related AEs with EUS-GE vs. ES in patients already treated by EUS-CDS for DMBO.

This finding is clinically relevant because clinicians and endoscopists must frequently deal with the double obstructive condition, both biliary and luminal, in patients affected by pancreatic or gastroduodenal cancers. It may be argued that, since duodenal stricture represents a risk factor for EUS-CDS dysfunction [24], this might be considered a suboptimal solution in case of concomitant biliary/luminal obstruction. This aspect was behind the aim of our study; nevertheless, waiting for conclusive evidence supporting different approaches, EUS-CDS is, to date, the first-line option for biliary drainage in case of ERCP failure according to international guidelines [25]. Thus, the need for facing GOO symptoms and a previously placed EUS-CDS is a common situation. 

In this setting, despite the spreading of an EUS-guided approach for GOO symptoms, it has been questioned if EUS-GE was a safe option in patients already treated with EUS-CDS for DMBO for the risk of a negative impact on biliary LAMS patency. The assumption behind this concern was that enteral stenting guarantees the “natural” passage of food and gastric material from the stomach through the second duodenal portion, theoretically preventing food stasis where the biliary LAMS is placed. On the other hand, EUS-GE, although effectively solving GOO symptoms, could lead to food stasis at the duodenal, pre-stenotic level, thus causing disfunction of the biliary LAMS and related AEs. 

Our study showed a comparable risk of biliary stent dysfunction, and/or biliary-related AEs, ruling out any detrimental effect of EUS-GE compared to ES. In both groups, most of the events seem to be due to stent obstruction (i.e., ascending cholangitis, jaundice recurrence), requiring endoscopic reintervention. If the biliary stent disfunction etiology (obstruction) was expected, the opportunity of managing them endoscopically in both the groups was an important finding. Nevertheless, despite in our series all the cases of stent dysfunction requiring only stent revision, and, eventually, coaxial double pig-tail plastic stent (DPPS) placement to be solved, we want to underline that EUS-CDS dysfunction may be a complex condition potentially requiring other EUS-guided interventions [26]

On the other hand, focusing on GOO symptoms, coherently with previous evidence [14,15], both EUS-GE and ES showed high technical and clinical success rates in our cohort. However, looking at stent patency, we confirmed the favorable trend previously reported in case of EUS-GE, with 0 cases of GOO symptoms relapse, suggesting more durable results compared with ES (six cases, 11.5%) even if not statistically significant (*p* = 0.16). This, coupled with the previously discussed lack of any adjunctive risk of biliary stent dysfunction, may further strengthen the role of EUS-GE even in this challenging subgroup of patients. It may be argued that surgical gastroenteroanastomosis may still be considered in fit patients in such conditions [27]. However, previous studies comparing endoscopic vs. surgical gastroenteroanastomosis showed equivalent clinical success rates, and better cost-effectiveness suggesting that the endoscopic approach should be preferred when expertise and devices are available [28,29,30].

Despite its strengths, our study has several limitations: the main drawback is its retrospective nature which may undermine our conclusions due to the risk of biases. However, our real-life experience reassures the safety profile of the endoscopic treatment of GOO even in this subgroup of patients and may be informative for designing future prospective comparative studies. Secondly, the relatively limited sample size prevents any statistical inference aimed at identifying any particular features related to better outcomes with either one or the other approach. Nevertheless, considering the literature on this topic is very limited, the choice to provide all technical features of the two procedures, might give the reader a useful insight in this challenging scenario. Furthermore, the multicenter setting may reassure of the reproducibility of our findings.

## 5. Conclusions

In conclusion, our results suggest that both ES and EUS-GE are safe and effective for the treatment of GOO in patients already treated with EUS-CDS for DMBO, with no difference AEs (8% vs. 17.3%), and biliary dysfunction risk (12.5% vs. 17.3%). Considering EUS-GE seems to provide more durable results (0% vs. 11.5%), it should be preferred over ES in experienced centers even in this subgroup of patients. 

## Figures and Tables

**Table 1:** Baseline characteristics.

BASELINE	GEA (25 pts)	Duodenal Stent (52 pts)	
Gender male, *n* (%)	13 (52%)	35 (67.3%)	0.19
Age	70 (59–81)	71 (66–75)	0.54
**Etiology** Pancreatic cancer, n (%)	17 (68%)	44 (84.6%)	0.34
Ampullary cancer, n (%)	1 (4%)	3 (5.7%)	
Duodenal cancer, n (%)	1 (4%)	1 (1.9%)	
Cholangiocarcinoma, n (%)	2 (8%)	1 (1.9%)	
Other, n (%)	4 (16%)	3 (5.7%)	
**Failed ERCP Reason** Failed cannulation in a reachable papilla, n (%)	2 (8%)	3 (5.7%)	0.97
Unreachable papilla, n (%)	4 (16%)	10 (19.2%)	
Duodenal stricture, n (%)	19 (76%)	38. (73%)	
Other, n (%)	0 (0%)	1 (1.9%)	
Technical success EUS-CDS, n (%)	25 (100%)	52 (100%)	1.0
Clinical success EUS-CDS, n (%)	25 (100%)	51 (98%)	0.74
AE EUS-CDS, n (%) (pre-GOO treatment)	1 (4%)	6 (11.5%)	0.42
GOOS Score, n (%)			
0	4 (16%)	11 (21.1%)	0.40
1	20 (80%)	32. (61.5%)	
2	1 (4%)	8 (15.5%)	
3	0 (0%)	1 (1.9%)	

**Table 2:** Outcomes.

OUTCOMES	EUS-GE (25 pts)	ES (52 pts)	
Days after EUS-CDS	5 (0–18)	1 (0–10)	0.55
AE	2 (8%) ^1^	9 (17.3%) ^2^	0.48
Severe AE	1 (4%)	4 (7.6%)	0.53
EUS-CDS dysfunction rate *	3/24 (12.5%)	9/52 (17.3%)	0.74
EUS-GE/ES dysfunction rate **	0/24 (0%)	6/52 (11.5%)	0.16
Re-intervention rate	3 (12.5%)	12 (23.1%)	0.36
Technical success	24/25 (96%)	52/52 (100%)	0.32
Clinical success	24/24 (100%)	51/52 (98%)	1.0
Average GOOS pre	0.88	0.99	0.14
Average GOOS post	2.6	2.5	0.65
Average improvement	1.71	1.6	0.46
Hospital stays, days (range)	3 (3–4)	5 (3–8)	0.15
Procedure duration (min)	30 (16–40)	25 (15–35)	0.20

^1^ Migration and bleeding. ^2^ three bleeding; one stent obstruction; five relapse of GOO. * Cholangitis, jaundice, biliary stent obstruction. ** Stent migration, stent obstruction, relapse of GOO symptoms.

## Data Availability

The data presented in this study are available in this article (and Supplementary Material).

## Supplementary Materials

The following supporting information can be downloaded at: https://www.mdpi.com/article/10.3390/cancers16193375/s1. Table S1. Procedural characteristics of enteral stents. Table S2. Procedural characteristic of EUS-GEA.

## References

[1] Ahmed O., Lee J.H., Thompson C.C., Faulx A. (2021). AGA Clinical Practice Update on the Optimal Management of the Malignant Alimentary Tract Obstruction: Expert Review. Clin. Gastroenterol. Hepatol..

[2] Nabi Z., Reddy D.N. (2019). Endoscopic Management of Combined Biliary and Duodenal Obstruction. Clin. Endosc..

[3] Law R.J., Chandrasekhara V., Bhatt A., Bucobo J.C., Copland A.P., Krishnan K., Kumta N.A., Pannala R., Parsi M.A., Rahimi E.F. (2021). Lumen-Apposing Metal Stents (with Videos). Gastrointest. Endosc..

[4] Giovannini M., Moutardier V., Pesenti C., Bories E., Lelong B., Delpero J. (2001). Endoscopic Ultrasound-Guided Bilioduodenal Anastomosis: A New Technique for Biliary Drainage. Endoscopy.

[5] Amato A., Sinagra E., Celsa C., Enea M., Buda A., Vieceli F., Scaramella L., Belletrutti P., Fugazza A., Cammà C. (2021). Efficacy of Lumen-Apposing Metal Stents or Self-Expandable Metal Stents for Endoscopic Ultrasound-Guided Choledochoduodenostomy: A Systematic Review and Meta-Analysis. Endoscopy.

[6] Parsa N., Nieto J.M., Powers P., Mitsuhashi S., Abdelqader A., Hadzinakos G., Anderloni A.A., Fugazza A., James T.W., Arlt A. (2020). Endoscopic Ultrasound-Guided Drainage of Pancreatic Walled-off Necrosis Using 20-Mm versus 15-Mm Lumen-Apposing Metal Stents: An International, Multicenter, Case-Matched Study. Endoscopy.

[7] Fugazza A., Colombo M., Repici A., Anderloni A. (2020). Endoscopic Ultrasound-Guided Gallbladder Drainage: Current Perspectives. Clin. Exp. Gastroenterol..

[8] Fugazza A., Fabbri C., Di Mitri R., Petrone M.C., Colombo M., Cugia L., Amato A., Forti E., Binda C., Maida M. (2022). EUS-Guided Choledochoduodenostomy for Malignant Distal Biliary Obstruction after Failed ERCP: A Retrospective Nationwide Analysis. Gastrointest. Endosc..

[9] Wannhoff A., Ruh N., Meier B., Riecken B., Caca K. (2021). Endoscopic Gastrointestinal Anastomoses with Lumen-Apposing Metal Stents: Predictors of Technical Success. Surg. Endosc..

[10] Fugazza A., Khalaf K., Spadaccini M., Facciorusso A., Colombo M., Andreozzi M., Carrara S., Binda C., Fabbri C., Anderloni A. (2024). Outcomes Predictors in Endoscopic Ultrasound-Guided Choledochoduodenostomy with Lumen-Apposing Metal Stent: Systematic Review and Meta-Analysis. Endosc. Int. Open.

[11] Espinel J., Vivas S., Muñoz F., Jorquera F., Olcoz J.L. (2001). Palliative Treatment of Malignant Obstruction of Gastric Outlet Using an Endoscopically Placed Enteral Wallstent. Dig. Dis. Sci..

[12] Adler D.G., Baron T.H. (2002). Endoscopic Palliation of Malignant Gastric Outlet Obstruction Using Self-Expanding Metal Stents: Experience in 36 Patients. Am. J. Gastroenterol..

[13] Martins R.K., Brunaldi V.O., Fernandes A.L., Otoch J.P., Artifon E.L.d.A. (2023). Palliative Therapy for Malignant Gastric Outlet Obstruction: How Does the Endoscopic Ultrasound-Guided Gastroenterostomy Compare with Surgery and Endoscopic Stenting? A Systematic Review and Meta-Analysis. Ther. Adv. Gastrointest. Endosc..

[14] Chen Y.-I., Itoi T., Baron T.H., Nieto J., Haito-Chavez Y., Grimm I.S., Ismail A., Ngamruengphong S., Bukhari M., Hajiyeva G. (2017). EUS-Guided Gastroenterostomy Is Comparable to Enteral Stenting with Fewer Re-Interventions in Malignant Gastric Outlet Obstruction. Surg. Endosc..

[15] Ge P.S., Young J.Y., Dong W., Thompson C.C. (2019). EUS-Guided Gastroenterostomy versus Enteral Stent Placement for Palliation of Malignant Gastric Outlet Obstruction. Surg. Endosc..

[16] Fabbri C., Scalvini D., Paolo G., Binda C., Mauro A., Coluccio C., Mazza S., Trebbi M., Torello Viera F., Anderloni A. (2024). Complications and Management of Interventional Endoscopic Ultrasound: A Critical Review. Best Pract. Res. Clin. Gastroenterol..

[17] von Elm E., Altman D.G., Egger M., Pocock S.J., Gøtzsche P.C., Vandenbroucke J.P., STROBE Initiative (2007). The Strengthening the Reporting of Observational Studies in Epidemiology (STROBE) Statement: Guidelines for Reporting Observational Studies. Lancet.

[18] Veitch A.M., Vanbiervliet G., Gershlick A.H., Boustiere C., Baglin T.P., Smith L.-A., Radaelli F., Knight E., Gralnek I.M., Hassan C. (2016). Endoscopy in Patients on Antiplatelet or Anticoagulant Therapy, Including Direct Oral Anticoagulants: British Society of Gastroenterology (BSG) and European Society of Gastrointestinal Endoscopy (ESGE) Guidelines. Endoscopy.

[19] Chen Y.-I., Kunda R., Storm A.C., Aridi H.D., Thompson C.C., Nieto J., James T., Irani S., Bukhari M., Gutierrez O.B. (2018). EUS-Guided Gastroenterostomy: A Multicenter Study Comparing the Direct and Balloon-Assisted Techniques. Gastrointest. Endosc..

[20] Rai P., Kumar P., Goel A., Singh T.P., Sharma M. (2023). Nasojejunal Tube-assisted Endoscopic Ultrasound-guided Gastrojejunostomy for the Management of Gastric Outlet Obstruction Is Safe and Effective. DEN Open.

[21] Troncone E., Fugazza A., Cappello A., Del Vecchio Blanco G., Monteleone G., Repici A., Teoh A.Y.B., Anderloni A. (2020). Malignant Gastric Outlet Obstruction: Which Is the Best Therapeutic Option?. World J. Gastroenterol..

[22] Pan Y., Pan J., Guo L., Qiu M., Zhang J. (2014). Covered versus Uncovered Self-Expandable Metallic Stents for Palliation of Malignant Gastric Outlet Obstruction: A Systematic Review and Meta-Analysis. BMC Gastroenterol..

[23] Cotton P.B., Eisen G.M., Aabakken L., Baron T.H., Hutter M.M., Jacobson B.C., Mergener K., Nemcek A., Petersen B.T., Petrini J.L. (2010). A Lexicon for Endoscopic Adverse Events: Report of an ASGE Workshop. Gastrointest. Endosc..

[24] Vanella G., Bronswijk M., van Wanrooij R.L., Dell’Anna G., Laleman W., van Malenstein H., Voermans R.P., Fockens P., Van der Merwe S., Arcidiacono P.G. (2023). Combined Endoscopic MAnagement of BiliaRy and GastrIc OutLET Obstruction (CABRIOLET Study): A Multicenter Retrospective Analysis. DEN Open.

[25] Dumonceau J.-M., Tringali A., Papanikolaou I.S., Blero D., Mangiavillano B., Schmidt A., Vanbiervliet G., Costamagna G., Devière J., García-Cano J. (2018). Endoscopic Biliary Stenting: Indications, Choice of Stents, and Results: European Society of Gastrointestinal Endoscopy (ESGE) Clinical Guideline—Updated October 2017. Endoscopy.

[26] Vanella G., Dell’Anna G., van Wanrooij R.L.J., Bronswijk M., Voermans R.P., Laleman W., van Malenstein H., Fockens P., Van der Merwe S., Arcidiacono P.G. (2024). Managing Dysfunctions and Reinterventions in Endoscopic Ultrasound-Guided Choledochoduodenostomy with Lumen Apposing Metal Stents: Illustrated Technical Review (with Videos). Dig. Endosc..

[27] Ojima T., Nakamori M., Nakamura M., Katsuda M., Hayata K., Yamaue H. (2017). Laparoscopic Gastrojejunostomy for Patients with Unresectable Gastric Cancer with Gastric Outlet Obstruction. J. Gastrointest. Surg..

[28] Fugazza A., Andreozzi M., Asadzadeh Aghdaei H., Insausti A., Spadaccini M., Colombo M., Carrara S., Terrin M., De Marco A., Franchellucci G. (2024). Management of Malignant Gastric Outlet Obstruction: A Comprehensive Review on the Old, the Classic and the Innovative Approaches. Medicina.

[29] Bomman S., Ghafoor A., Sanders D.J., Jayaraj M., Chandra S., Krishnamoorthi R. (2022). Endoscopic Ultrasound-Guided Gastroenterostomy versus Surgical Gastrojejunostomy in Treatment of Malignant Gastric Outlet Obstruction: Systematic Review and Meta-Analysis. Endosc. Int. Open.

[30] Perez-Miranda M., Tyberg A., Poletto D., Toscano E., Gaidhane M., Desai A.P., Kumta N.A., Fayad L., Nieto J., Barthet M. (2017). EUS-Guided Gastrojejunostomy Versus Laparoscopic Gastrojejunostomy: An International Collaborative Study. J. Clin. Gastroenterol..

