# Supplementary material for: Palliation of Gastric Outlet Obstruction in Case of Biliary Obstruction—A Retrospective, Multicenter Study: The B-GOOD Study
Source: Cancers (Basel). 2024 Oct 2;16(19):3375. doi: 10.3390/cancers16193375 (PMC11475973; doi:10.3390/cancers16193375)
Supplement: Supplementary file 1 [file cancers-16-03375-s001.zip › cancers-3165141-supplementary.pdf]

cancers MDPI

Supplementary Materials

Palliation of Gastric Outlet Obstruction in Case of Biliary Obstruction—A Retrospective, Multicenter Study: The B-GOOD Study

Table S1. Procedural characteristics of enteral stents.

Characteristic	Value (%)
**Length**	
60mm	26 (50%)
90mm	19 (36,5%)
100mm	3 (5,67%)
120mm	4 (7,69%)
**Diameter**	
18mm	3(5,77%)
20mm	6 (11,54%)
22mm	42 (80,67%)
25mm	1 (1,92%)
**Proximal position**	
Transpyloric	13 (25%)
D1 covering EUS-CDS	5 (9,62%)
D1 not covering EUS-CDS	22 (42,31%)
D2	12 (23,08%)

Table S2. Procedural characteristic of EUS-GEA.

Characteristic	Value (%)
**Stent Type**	
Hot-Axios	26 (100%)
**Stent diameter**	
10mm	1 (4%)
15mm	4 (16%)
20mm	20 (80%)
**EUS-GE technique**	
Balloon	8 (32%)
Free-hand	8 (32%)
Jejunal catheter	9 (36%)

**EUS-GE access**	
Single stage	25 (100%)
**EUS-GE fluid infusion**	
Combination	16 (64%)
Methylen blue	9 (36%)
